# The shadow of fear: hate crime victimization and stress after Charlottesville

**DOI:** 10.3389/fpsyg.2024.1384470

**Published:** 2024-06-27

**Authors:** Joshua Hellyer, Johanna Gereke

**Affiliations:** Mannheim Centre for European Social Research, University of Mannheim, Mannheim, Germany

**Keywords:** hate crimes, stress, African-Americans, racial and ethnic minorities, Charlottesville, United States, natural experiment

## Abstract

**Introduction:**

Recent years have witnessed an increase in highly publicized attacks targeting members of ethnoracial and religious minority groups. To date, existing research has primarily focused on the tendency for such “trigger events” to generate violent aftershocks. We argue that beyond such ripple effects, highly salient trigger events significantly increase hate-crime related stress among racial and ethnic minorities. Additionally, we explore whether these effects are limited to the group most clearly targeted, or if they “spill over” to other minoritized communities.

**Methods:**

To study reactions to hate crimes, we draw upon national survey data (*N* = 1,122) in combination with a natural experiment involving the Unite the Right rally and vehicle attack in Charlottesville, Virginia in August 2017. We employ an “unexpected event during survey” design to estimate the causal effect of the Charlottesville rally on stress about hate crimes.

**Results:**

We first show that there was an increase in anti-Black hate crimes in the 2 weeks following the Charlottesville incident. We also find a corresponding increase in stress due to the perception of personal vulnerability to hate crimes among African-Americans. However, we do not observe a significant increase in levels of stress following the trigger event among Hispanics and Asian Americans.

**Discussion:**

Our results suggest that highly publicized instances of intergroup violence can have significant impacts on stress about hate crime victimization within the target group. However, we find that this effect is short-lived, and that both violent aftershocks and the general climate of fear spurred by hate crimes may be racially bounded.

## 1 Introduction

Recent years have witnessed an alarming increase in highly publicized attacks against members of minority groups. A unique convergence of factors including the COVID-19 pandemic (Gray and Hansen, 2021; Dipoppa et al., 2023; Han et al., 2023), mass migration (Stacey et al., 2011; Piatkowska et al., 2020), and the resurgence of far-right political movements (Devine, 2021; Müller and Schwarz, 2021) have led to devastating attacks on marginalized communities in a growing number of communities across the world including Atlanta, USA; Christchurch, New Zealand; and Hanau, Germany. To date, scholarship about such attacks has primarily focused on the tendency for such “trigger events” to generate violent aftershocks. However, we argue that these events generate a climate of fear and may have broader negative consequences for minority communities. In particular, we hypothesize that trigger events increase stress related to being affected by a hate crime.

The idea that an attack on a specific group may cause fear and anxiety in other members of the group is not entirely new. Previous research has shown that exposure to violence against one's group can have negative impacts on mental health (Curtis et al., 2021; Eichstaedt et al., 2021), and lead people to change their behavior to avoid victimization (Perry and Alvi, 2012; Paterson et al., 2019a; Walters et al., 2020). Furthermore, this has become a common narrative in reporting about episodes of targeted racial violence across the world. Reporting on a 2022 shooting in Buffalo, New York targeting Black shoppers at a supermarket, a *BBC* headline read: “Buffalo shooting: Black Americans describe grief and fear” (Tawfik, 2022), and regarding an attack against Muslims, the *New York Times* reported: “Killings of 4 Men in Albuquerque Leave Muslim Community in Fear” (Bohra and Patel, 2022). A shortcoming of this media framing of violent events and of prior research is that it remains unclear whether such attacks also affect members of other marginalized groups. If members of other ethnic groups are also negatively impacted by such violence, then this framing, as well as past research, may significantly underestimate the impact of these events. These impacts may be wide-ranging, from individual consequences like stress and perceived vulnerability to societal consequences like health care expenditures, as well as deteriorating intergroup relations and social cohesion. We will address this research gap by assessing how members of three different ethnoracial minority groups respond to a single trigger event.

In this study, we specifically study the effect of the Unite the Right rally and vehicle attack in Charlottesville, Virginia. On August 12, 2017, torch-bearing white supremacists marched through the streets of Charlottesville before driving a car into a crowd of counter-protesters, killing one and injuring dozens of others. Like other violent crimes motivated by bias, the rally was not merely intended to victimize any one individual, but to threaten the wider community. However, the Charlottesville rally is somewhat unique in that this community may be a rather broad group. Rather than targeting a single racial or ethnic group, the rally and vehicle attack were meant to support a broad white nationalist agenda that opposes not only Black Americans, but also Jews, Muslims, immigrants, the LGBTQ+ community, and the political left. Thus, relative to an attack like the Buffalo shooting, which explicitly targeted Black Americans, it is less clear which groups will feel targeted by this blatant expression of white supremacist ideology.

We study stress about hate crimes using the Stress in America survey, a large nationally representative survey with large samples of racial and ethnic minorities. Fieldwork was conducted throughout the month of August 2017, uniquely allowing us to employ an unexpected event during survey analysis to examine the effects of the Charlottesville rally on levels of stress among African-Americans as well as Hispanics and Asian Americans. To preview our findings, we find that the Charlottesville attack resulted in an increase in stress about personal hate crime victimization among African-Americans. Interestingly, these results are observed only among African-American respondents, suggesting that both the surge in violent aftershocks and the general climate of fear may be racially bounded.

## 2 Hate crime and its impact on minority wellbeing

In U.S. law, a hate crime is defined as a criminal action taken by an offender due to their bias against another's race, ethnicity, religion, disability, sexual orientation, gender, or gender identity. Any crime motivated by prejudice could thus be classified as a hate crime, from property crimes like vandalism to violent crimes like arson, assault, or murder. Scholars have defended the government's special interest in hate crimes by asserting that hate crimes pose additional harms above and beyond those normally associated with non-bias crimes (Levin, 1999; Iganski, 2001; Cogan, 2002). Not only do bias crimes pose greater risk of physical and psychological harm to victims than non-bias crimes (Fetzer and Pezzella, 2019; Lantz and Kim, 2019), but they also send a message to the wider community that members of targeted groups are not welcome, and are not safe (Iganski, 2001; Schwitter and Liebe, 2024). This symbolic harm and its potential to damage intergroup relations have led social psychologists to consider hate crime as a distinct form of aggression (Craig, 2002).

Researchers have found that even target group members who only hear of hate crimes second-hand still suffer from increased anger and anxiety and are likely to change their behavior to avoid victimization (Perry and Alvi, 2012; Paterson et al., 2019a,b; Walters et al., 2020). However, evidence for the effects of hate crime on wellbeing remains limited, relying primarily on small and non-random samples. The few studies that have assessed this connection quantitatively have focused primarily on the LGBT community (Paterson et al., 2019a; Walters et al., 2020), which may respond to crimes in a different way than members of more visible minority groups who might be more easily targeted (Chongatera, 2013). Furthermore, there may also be meaningful heterogeneity within groups with respect to the personal impact of hate crime (Maduro et al., 2020; Ponce et al., 2022). There is also some recent evidence that the effects of hate crime might extend beyond the targeted group: a higher rate of hate crimes in a community is associated with higher overall levels of hypertension, diabetes, and obesity among all residents regardless of race (Gero et al., 2022). Thus, we still do not understand the boundary conditions for the effect of second-hand hate crime victimization on minority members' health and wellbeing.

### 2.1 Reactions to trigger events and spillover effects among minority respondents

A common theme in the hate crime literature is the temporal clustering of hate crime events. It is now well established that hate crime rates spike in the days following “trigger events” that create intergroup grievances (King and Sutton, 2013). Terror attacks have been the most commonly studied trigger event, with researchers finding significant increases in hate crimes following attacks in the U.S. (Disha et al., 2011; Deloughery et al., 2012; King and Sutton, 2013), the U.K. (Hanes and Machin, 2014; Devine, 2021; Piatkowska and Stults, 2022), and mainland Europe (Borell, 2015; Jacobs and van Spanje, 2021). Notably, these attacks need not be local to have an effect, with several European studies showing an increase in anti-Muslim hate crimes after the 9/11 attacks in the U.S. (Hanes and Machin, 2014; Borell, 2015).

Scholars have suggested two theoretical explanations for responses to violent trigger events. Most studies have focused on the responses of majority group members (i.e., hate crimes or negative attitudes toward minority groups) to attacks conducted by minority group members (Hopkins, 2010; Legewie, 2013; Hanes and Machin, 2014; Borell, 2015; Jungkunz et al., 2019; Frey, 2020; Jacobs and van Spanje, 2021; Cozzani et al., 2022). This dynamic of an “upward” attack by a minority group followed by “downward” attacks from the majority suggests a pattern of retaliation (Deloughery et al., 2012). However, this dynamic does not explain responses to attacks conducted by majority group members against minority groups, which have also been linked to increases in anti-minority violence (Esser and Brosius, 1996; Levin, 1999; Jäckle and König, 2017). In these situations, scholars have suggested that media reports of attacks can offer an example for other would-be perpetrators, spurring copycat attacks in what is sometimes called a “contagion” effect (Esser and Brosius, 1996). This effect may operate through social norms, as one attack against a certain group may signal that such behavior is socially acceptable (Álvarez-Benjumea and Winter, 2018; Bauer et al., 2018).

A small but growing literature evaluates how minority group members respond to trigger events targeting minority communities and the threatening environment they create, and how such threats affect minority stress, mental health and wellbeing. Research from the U.S. finds that Black Americans report poorer mental health after highly public instances of anti-Black violence (Curtis et al., 2021; Eichstaedt et al., 2021). European research finds that refugees showed similar declines in mental health after both exposure to anti-refugee attacks (Graeber and Schikora, 2021) as well as terrorist attacks attributed to foreigners (Frey, 2022).

One possibility by which trigger events might impact minorities' stress, wellbeing and health is by increasing the salience of group boundaries. Research on terrorist attacks finds that such incidents can reinforce the boundary between the victimized group and the group to which the perpetrator belongs (Jović et al., 2023). Drawing on the framework of Intergroup Emotions Theory (Mackie et al., 2008), heightened salience of group boundaries may lead individuals to identify more strongly with a group, spurring collective emotional responses. In the context of hate crimes, this suggests that members of the victim's group may experience increased stress about potential victimization. Exactly this response was found in a study of anti-LGBT hate crimes (Paterson et al., 2019a), but research has not yet examined whether similar reactions are found among racial or ethnic groups, nor whether these reactions extend to other minoritized groups.

Such an effect could be conceptualized as a negative, indirect form of the “secondary transfer effect” proposed by Pettigrew (2009) (see review by Vezzali et al., 2021). Some evidence for the negative form of this effect already exists, suggesting that negative contact experiences with a specific outgroup may lead to negative attitudes toward other outgroups as well (Meleady and Forder, 2019; Henschel and Derksen, 2023). A few studies have examined the possible spillover effects of trigger events with respect to prejudice, finding that Jihadist terror attacks led to increased prejudice toward other non-Muslim immigrant groups (Branton et al., 2011; Rousseau et al., 2011; McConnell and Rasul, 2021; Czymara and Gorodzeisky, 2024) and that the outbreak of COVID-19 also yielded significant discrimination against non-Asian minorities in the U.S. despite widespread attribution of the pandemic to China (Wenger and Lantz, 2022). This evidence suggests that trigger events may have a widespread impact on minority group members beyond those held responsible for these instances of intergroup conflict.

It is however not yet clear whether members of other groups perceive this “spillover,” i.e., whether they feel that they are at a higher risk of victimization due to the trigger event despite not belonging to the primary target group. However, an attack on another minority community might create conditions that have been associated with interethnic solidarity by strengthening a sense of exclusion from the majority (Pérez et al., 2023). Furthermore, other research shows that people tend to believe that prejudice against one group is correlated with prejudice against other groups, in what has been termed the “lay theory of generalized prejudice” (Chaney and Forbes, 2023). This suggests that minority group members may see the demonstrators in Charlottesville as likely to be prejudiced against their own groups as well, leading them to also fear the effects of increased hate.

## 3 Context: Unite the Right rally

To study the impact of trigger events on minority stress and hate crime victimization, we will focus on a single trigger event: the Unite the Right rally in Charlottesville, Virginia. On the night of August 11, 2017, groups of white supremacists carrying torches as well as Confederate, Nazi and Ku Klux Klan paraphernalia marched in the streets of Charlottesville. The rally was dispersed on the morning of August 12 after a white supremacist drove his car into a crowd of peaceful counter-protesters, killing 32-year-old Heather Heyer and injuring 35 others. This attack followed other instances of violence, including a group attack on an unarmed Black man in a parking garage as well as various reports of hand-to-hand combat and gunfire (Blout and Burkart, 2023). While the rally was ostensibly a protest of the removal of a statue of Confederate General Robert E. Lee, it also served a symbolic purpose to “mourn and resist a perceived cultural erosion of whiteness” (Perry, 2018) and was carefully planned to recruit new believers to the cause of white supremacy (Blout and Burkart, 2023). Following the rally, media attention was also focused on then-President Donald Trump's reaction to the incident, in which he blamed violence on “both sides” for Heyer's death (Perry, 2018). This assertion of violence conducted by anti-racist counter-protesters may have undermined public support for their cause, and driven additional support to white nationalist groups (Simpson et al., 2018). The rally has frequently been cited as a turning point for the “alt-right,” leading to greater awareness of the movement and its white nationalist ideology (Atkinson, 2018; van der Vegt et al., 2021).

Given the extensive media coverage of the event, the rally in Charlottesville represents a highly visible manifestation of white supremacy in America. This motive distinguishes this event from most other trigger events studied, in that it was conducted by members of the majority (White) population against minority groups. It is thus simultaneously a trigger event and a hate crime itself. While the rally was mainly an expression of white supremacy rather than a focused attack on a single outgroup, the event seems to have particularly targeted African-Americans and Jewish people. Not only were Black and Jewish local officials as well as residents of primarily Black neighborhoods targeted for harassment before the rally (Blout and Burkart, 2023), but anti-Semitic and anti-Black language was used frequently during the rally itself (Szilágyi, 2017).

Perhaps due to the blatant anti-Black rhetoric used, or the underlying debate about slavery and Confederate-era monuments, the rally seems to have negatively affected African-Americans in the Charlottesville area. Local emergency rooms reported an increase in African-American patients with symptoms of anxiety in the weeks following the event (Stephens, 2018), and in interviews, some local Black and multiracial youth reported feeling fear and a need for vigilance after the attack (Williams et al., 2021).

However, because these studies both focus on the local area, we do not know whether the rally had an effect on stress nationwide. We also know little about how members of other marginalized racial and ethnic groups responded to the attack. While white supremacist discourse is often focused on Black and Jewish people, it also demonizes a wide array of groups that are seen to oppose the views of white supremacists, including other religious, ethnic, and racial minorities; the LGBTQ+ community; and political opponents (Rieger et al., 2021). Members of any of these groups may thus also feel targeted by a visible expression of white supremacy. The present research will address both of these unanswered questions, offering information about stress levels among a larger and more diverse sample of minority group members from a wider geographic area.

## 4 Hypotheses

Addressing the gaps in the existing literature, we propose two central hypotheses about the impact of highly publicized instances of violence toward minorities on stress and hate crime victimization. Several considerations lead us to expect that the Unite the Right rally will increase hate crime-related stress among Black respondents specifically. While the rally did not exclusively target African-Americans, it originated from a debate over Confederate monuments and anti-Black rhetoric and imagery was widely used, thus Black respondents may feel particularly targeted. In this way, Charlottesville resembles other highly publicized instances of anti-Black violence, which have been shown to have negative impacts on mental health among African-Americans (Curtis et al., 2021; Eichstaedt et al., 2021). This is in line with some evidence finding that African-Americans in the Charlottesville area reported increased anxiety following the rally (Stephens, 2018).

**Hypothesis 1**: The events in Charlottesville will lead to an increase in hate crime-related stress among Black respondents.

We also aim to determine whether such an increase in stress was limited to members of one racial group, namely African-Americans, or whether the effects of the rally were felt more broadly among ethnic minority communities. Even though the rally prominently featured anti-Black rhetoric and imagery, the car attack did not target members of a specific group but rather a racially and ethnically diverse crowd of protesters. Given that the attack was a broad expression of white supremacy, an ideology that demonizes a wide array of minority ethnic, racial, and religious groups (Rieger et al., 2021), and that people assume that prejudices against various groups are co-occurring (Chaney and Forbes, 2023), we expect that all individuals belonging to ethnic minority groups will report greater hate crime-related stress following the event.

**Hypothesis 2**: Hate-crime related stress will be significantly higher following the hate crime at the Unite the Right Rally in Charlottesville among all non-white respondents (“spillover hypothesis”).

## 5 Hate crime in the aftermath of Charlottesville

Because we are interested in evaluating changes in hate crime-related stress, we conduct a preliminary analysis to evaluate whether the attacks in Charlottesville caused an increase in hate crime against the groups studied here. To do so, we use national statistics from the Federal Bureau of Investigation's (FBI) Uniform Crime Reporting (UCR) dataset for the years 2012–2017. The UCR has collected data on crimes motivated by bias since the 1990 passage of the Hate Crime Statistics Act, currently including biases based on race, religion, disability, sexual orientation, ethnicity, gender, and gender identity.

It is important to note that the figures reported by the UCR are likely to represent a conservative estimate of the actual number of hate crimes for several reasons. First, reporting to the federal database is optional at the federal level for all but federal law enforcement agencies. At the state level, only 30 states require that local and state agencies collect and report data about hate crimes. Second, even when reporting is required, the attribution of bias to a crime is somewhat subjective, and officers may be reluctant to do so due to factors like conflicting stories, political considerations, or lack of resources to investigate further (Balboni and McDevitt, 2001; McVeigh et al., 2003). Finally, hate crime victims may choose not to report these offenses due to sometimes tense relations between minority communities and the police, and victims' belief that their report will not be taken seriously (Balboni and McDevitt, 2001; Zaykowski, 2010; Pezzella et al., 2019).

**Figure 1:**
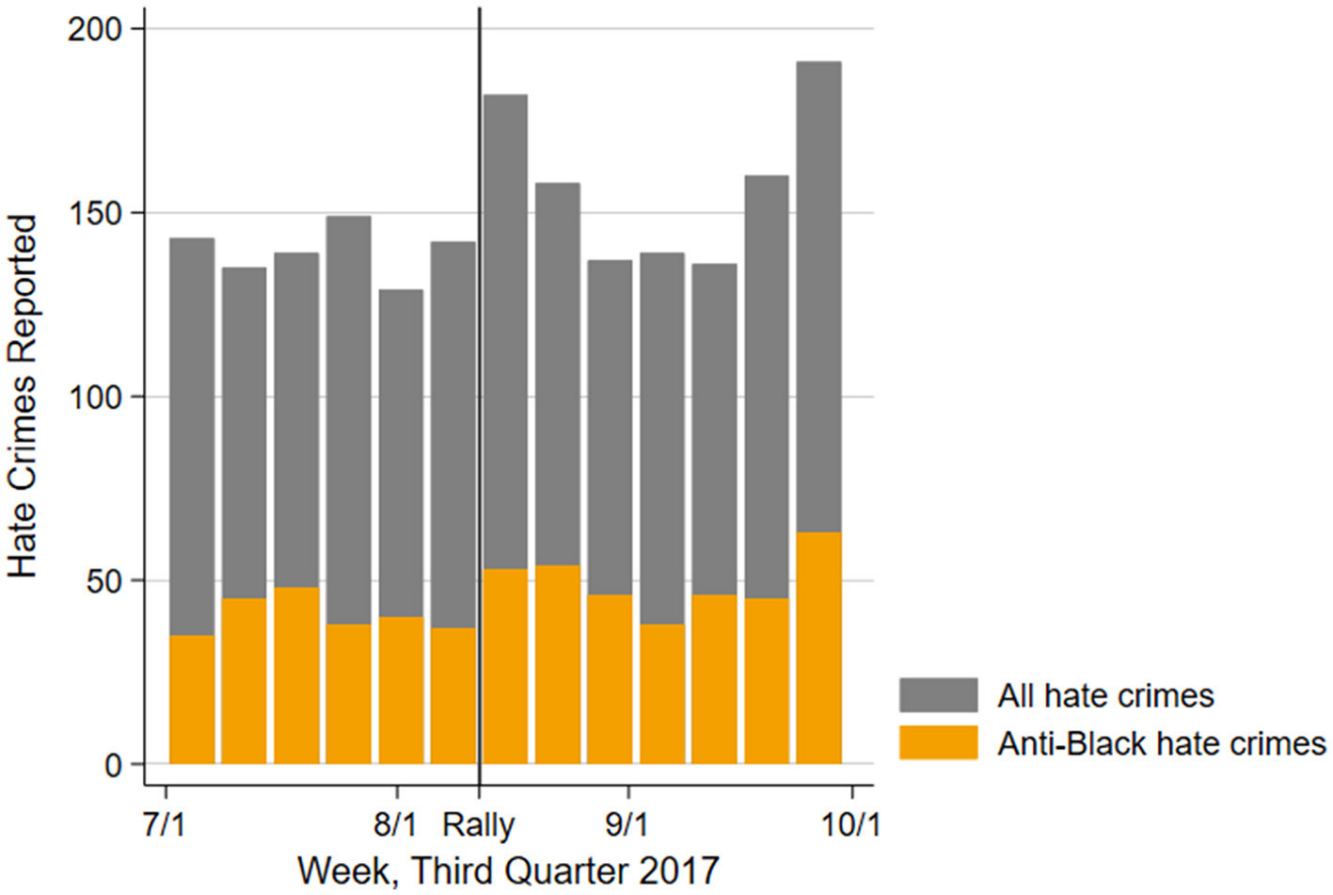
Number of hate crimes reported per week nationwide in the 3rd quarter of 2017 by target group, from FBI UCR data.

As a first indication of the effect of the Unite the Right rally on the number of hate crimes, we present an overview of the number of crimes reported by week and target group in Figure 1. The number of crimes targeting Black people spikes sharply in the week following the rally, returning to roughly pre-rally levels by 2 weeks later. In supplemental analyses presented in Supplementary Tables S2, S3, we demonstrate that the increase in anti-Black hate crimes is statistically significant within 2 weeks of the rally.^1^ However, there does not seem to be a large change in the number of hate crimes committed against Latine or Asian victims at the time of the rally. It is also important to note that the increase in crimes reported may be due to increased sensitivity following the rally, and thus it is not possible to separate differences in reporting behavior and differences in the number of hate crimes with this dataset.

## 6 Data and methods

### 6.1 Data: Stress in America survey

Having established that the events in Charlottesville caused an increase in hate crime targeting African-Americans, we next examine whether the events in Charlottesville increased hate crime-related stress for members of minority racial and ethnic groups. To do this, we use data from the 2017 edition of the Stress in America survey, conducted annually by the Harris Poll on behalf of the (American Psychological Association, 2017). The online survey, available in English and Spanish, asked 3,440 U.S. residents 18 and older about stressors in their lives and how they cope with stress. Respondents had previously agreed to participate in the Harris Poll's online panel. Because we are interested in the effects of Charlottesville on ethnoracial minority groups, we exclude White respondents.^2^ We also exclude Native American respondents due to a small sample size, leaving us with a sample entirely consisting of Black, Asian, and Hispanic adults.^3^ While religious minorities (especially Jews) may also feel increased stress following the rally (Hobbs et al., 2023), we lack information about religious affiliation in this dataset. For our analyses, we further restrict our sample to those respondents who answered the survey within one week of the Unite the Right rally (between August 5 and 19), excluding those who responded on August 12 itself (to reduce uncertainty about exposure to the treatment), yielding an analytic sample of 1,122 adults. A full description of the sample is available in Table 1. Note that our analytic sample differs from national demographics (taken from the 2017 American Community Survey) in several ways. In the Black and Hispanic samples, the respondents are more likely to be female, highly educated and not participating in the labor force. The Asian sample is closer to national averages, although still slightly more female and more highly educated.

**Table 1:** Sample descriptive statistics and comparison with American Community Survey (ACS) benchmarks.

	**Analytic sample**	**Full sample**	**ACS benchmark**
**Black respondents**	***N*** **= 437**	***N*** **= 775**	–
Male	0.30	0.30	0.47
Age	49.45	46.94	44.36
Education:			
No highschool	0.04	0.04	0.13
Highschool graduate	0.24	0.26	0.41
Some college	0.45	0.40	0.28
BA or above	0.27	0.30	0.18
Employment status:			
Employed	0.45	0.50	0.57
Unemployed	0.11	0.10	0.07
Not in labor force	0.43	0.40	0.36
Sexual minority	0.05	0.05	–
Stressed by hate crime	0.17	0.18	–
**Hispanic respondents**	***N*** **= 452**	***N*** **= 768**	**–**
Male	0.30	0.33	0.50
Age	43.47	42.96	41.07
Education:			
No highschool	0.08	0.07	0.28
Highschool graduate	0.38	0.32	0.37
Some college	0.34	0.36	0.22
BA or above	0.20	0.25	0.13
Employment status:			
Employed	0.49	0.52	0.65
Unemployed	0.10	0.09	0.05
Not in labor force	0.41	0.39	0.30
Sexual minority	0.08	0.08	–
Stressed by hate crime	0.13	0.14	–
**Asian respondents**	***N*** **= 233**	***N*** **= 486**	**–**
Male	0.42	0.40	0.47
Age	41.90	41.18	44.55
Education:			
No highschool	0.02	0.01	0.10
Highschool graduate	0.15	0.14	0.22
Some college	0.30	0.29	0.19
BA or above	0.53	0.55	0.48
Employment status:			
Employed	0.65	0.60	0.63
Unemployed	0.06	0.07	0.03
Not in labor force	0.29	0.32	0.34
Sexual minority	0.06	0.07	–
Stressed by hate crime	0.12	0.13	–

#### 6.1.1 Outcome variable: hate crime-related stress

As an outcome variable we study agreement with the statement: “I am stressed about personally being affected by hate crime.” Respondents were prompted to consider a variety of threats including terrorism, police violence, and gang violence, and respond that they did or did not cause them stress, yielding a binary measure. In another question, they were asked to consider whether the amount of hate crime nationally caused them stress, which we will also employ as a robustness check.

#### 6.1.2 Control variables

We also include a variety of control variables from the Stress in America survey in our regressions to account for the role of demographic factors in determining hate crime-related stress. To this end, we include race (Black, Hispanic, or Asian), sex (male or female, excluding those of other sexes due to small *N*), age, educational attainment, employment status, political party preference (Democrat, Republican, independent, or other), and sexual orientation (heterosexual or non-heterosexual). We also use respondents' self-reported ZIP codes in combination with 2017 American Community Survey 5-year population estimates to determine the percentage of the residents in a respondent's ZIP code who belong to the same racial group. Prior research suggests that hate crimes are less likely in communities with larger numbers of coethnics, as there is less opportunity for intergroup contact of any kind (Disha et al., 2011; Piatkowska et al., 2019; Rees et al., 2019). None of the respondents in our sample are missing data for the outcome variable, or for any of the control variables except for sexual orientation. The 50 respondents who declined to give sexual orientation information are handled via listwise deletion.

### 6.2 Method: unexpected event during survey design as causal identification strategy

**Figure 2:**
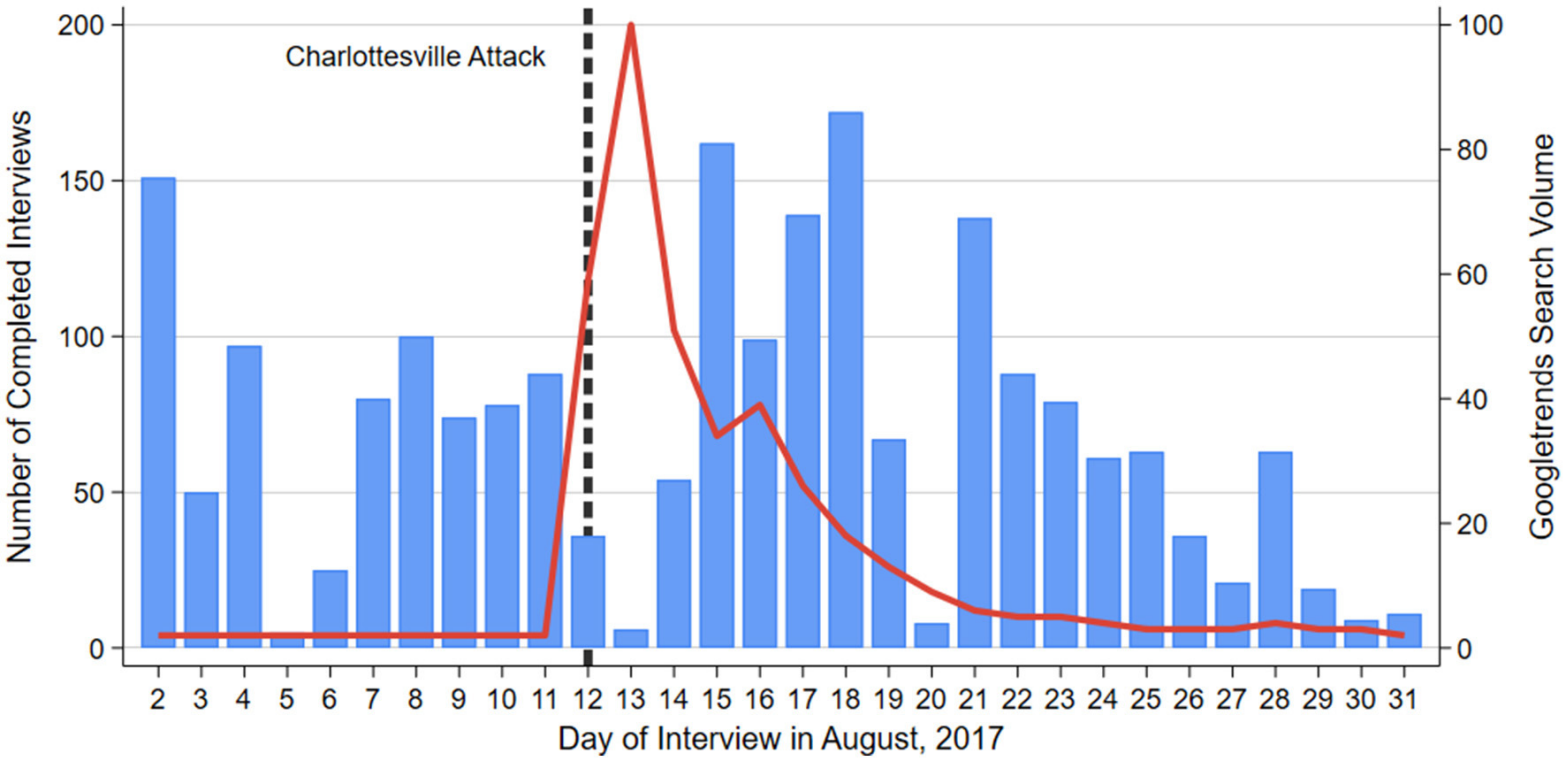
Number of responses to the Stress in America survey (blue) and Google search volume for “Charlottesville” (red) by date.

The Stress in America dataset is well-suited to our research question not only because it includes information about stressors and a variety of control variables, but also because it was fielded throughout the month of August 2017 (August 2–31) when the Unite the Right rally took place.^4^ This allows us to use an “Unexpected Event during Survey Design” (UESD) (Muñoz et al., 2020) to estimate the causal effect of the events in Charlottesville on hate crime-related stress levels among Black, Hispanic, and Asian U.S. adults. We thus compare responses before and after August 12 (the date of the car attack) to determine if respondents were more concerned about hate crime after the rally. Our estimates may reflect a conservative estimate of the rally's effects, as few surveys were completed on the weekend immediately following the rally (on Friday night into Saturday morning) when Google search interest reached its peak (indicated in red in Figure 2). We would expect the rally to have the strongest effects on these dates, even as search interest in Charlottesville remained high throughout the week studied.

We believe that a UESD design is appropriate for this event and this dataset for several reasons. First, as shown in blue in Figure 2, a sizable number of surveys were completed before and after Charlottesville, allowing for a meaningful comparison between treatment and control groups. Second, as indicated by the red line, there is no evidence of an anticipation effect. Search interest in “Charlottesville” rose dramatically on August 12 and 13 before slowly returning to roughly pre-rally levels by the end of the month. This suggests that the event was unexpected, and despite the fact that the rally was planned in advance, few would have been aware of it prior to the widespread news coverage after the car attack. Third, balance tests reveal that pre-rally respondents do not differ dramatically from post-rally respondents in a variety of demographic variables, as shown in Supplementary Tables S4–S7.

## 7 Main results: stress after Charlottesville

Our main results from this UESD analysis are presented in Table 2. We present three models that vary in the number of controls added, all of which are estimated as linear probability models because our analyses include interaction terms (Mood, 2010). In Model 1, we estimate the effect of the Charlottesville rally on all respondents' level of hate crime-related stress with no controls, finding only a small and insignificant result. In Model 2, we include respondents' race as well as interactions of race and the rally date in order to determine whether the effect of the rally may vary by race. We find evidence that it does: the overall effect of the rally becomes larger and significant, and we find that the rally has a significant effect increasing hate-crime related stress for Black Americans (in support of H1). However, we do not find any evidence that following the Charlottesville incident, Hispanic or Asian American respondents experienced higher levels of hate-crime related stress (H2 “spillover hypothesis” is not supported).

**Table 2:** Stressed about personally being affected by hate crime.

	**(1)**	**(2)**	**(3)**
After Charlottesville	0.03	0.09^*^	0.10^**^
	(0.02)	(0.04)	(0.04)
Black		(ref.)	(ref.)
Hispanic		0.02	0.03
		(0.04)	(0.04)
Asian		0.02	0.03
		(0.04)	(0.05)
After × Hispanic		−0.10^*^	−0.10^*^
		(0.05)	(0.05)
After × Asian		−0.11^+^	−0.12^*^
		(0.06)	(0.06)
Male			0.00
			(0.02)
Age			−0.00
			(0.00)
Education (9 pt)			−0.01^*^
			(0.01)
Unemployed			−0.01
			(0.04)
Not in labor force			−0.04^+^
			(0.02)
Party: Republican			−0.09^**^
			(0.03)
Party: Independent			−0.05
			(0.03)
Party: Other			−0.07^*^
			(0.03)
Perc. Own Race in Zipcode			−0.09^*^
			(0.04)
Sexual minority			0.08
			(0.05)
Constant	0.13^***^	0.12^***^	0.28^***^
	(0.02)	(0.03)	(0.05)
*N*	1122	1122	1122

Unstandardized regression coefficients with standard errors in parentheses. ^*^p < 0.05. ^**^p < 0.01. ^***^p < 0.001. ^+^*p* < 0.1.

**Figure 3:**
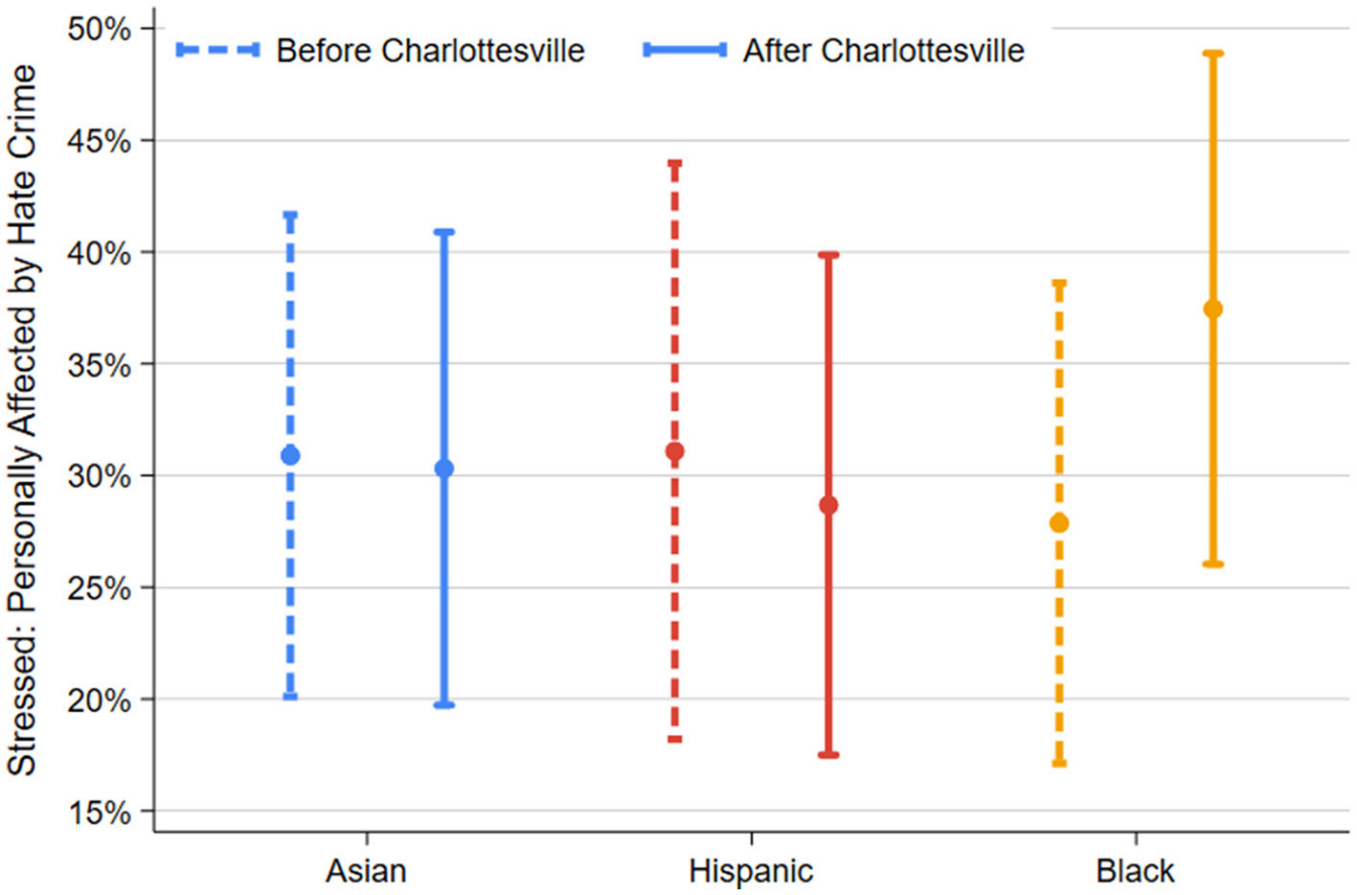
Percent stressed about personally being affected by a hate crime before and after 12 August 2017 by ethnoracial group. Error bars indicate 95% confidence intervals. Full regression results are shown in Table 2, Model 3.

In Model 3, we add a variety of demographic controls as described above. These results are also presented graphically in Figure 3. Our findings here are broadly similar to Model 2, although some control variables have significant negative effects on stress. Respondents who identified as Republicans or reported a political affiliation of “Other” reported less stress than Democrats (the comparison group). Additionally, respondents who lived in a community with a higher percentage of co-ethnics reported less hate crime-related stress.

In addition to the balance tests described above, we test the robustness of these results in various ways, as suggested by Muñoz et al. (2020). Full results of these tests are available in Appendix, but we will briefly describe each test here. First, we run placebo tests in which we estimate the effect of two other dates (August 7 and 27). As shown in Supplementary Table S8, we find no significant effect at either of these dates. Second, we run the same analyses on three alternative versions of the sample: one including all Black, Hispanic, and Asian respondents (even those who responded more than a week before or after the rally), one excluding Republican respondents, and one also including White respondents. We find similar trends in the non-Republican sample (Supplementary Table S10), the sample including White respondents (Supplementary Table S11), and in the full sample (Supplementary Table S9), although the effect of the rally is no longer significant in the full sample. Next, we test an alternative dependent variable on our original sample, using respondents' stress about hate crime as a national issue rather than their stress about being personally affected by hate crime. Results in Supplementary Table S12 indicate broadly similar trends, except that effect sizes are larger and that men are less likely to report stress about national levels of hate crime. We also apply entropy balancing to our data using the “ebalance” package in Stata. Entropy balancing is a strategy that constructs and applies a set of matching weights to ensure balance between treatment and control conditions on all covariates included in the model (Hainmueller, 2012). Again, we find that the rally has a significant positive effect on levels of stress only among Black respondents, as shown in Supplementary Table S13. Finally, as a falsification test, we run our main analyses on two variables indicating stress about money and the economy, variables that should be unaffected by the rally. As shown in Supplementary Table S14, we find no significant effect of the rally on financial stress, suggesting that changes in stress are due to Charlottesville and not other co-occurring factors. Altogether, these tests indicate that the assumptions of our UESD setup seem to hold in this case, and that our results are robust to a variety of specifications.

Another threat to a UESD analysis is the possibility that not all respondents are aware of the event, although this should be unlikely for an event like the Unite the Right rally which was widely discussed on social media and featured in international headlines. Only 4% of respondents in our sample report never watching the news, and only eight respondents report never watching the news or using social media, providing some evidence that the vast majority should be aware of the event. As an additional test of compliance, we also examine whether respondents report that the political climate is a significant stressor (a binary variable, yes or no) in Supplementary Table S15. Here, we see a large and significant effect of the rally that does not differ across racial and ethnic groups. The finding that this effect is particularly pronounced among Democrats, a group that may find the rally especially troubling, offers an additional signal that the events in Charlottesville are salient to the Stress in America respondents.

## 8 Discussion and conclusion

It has long been suggested that minority group members suffer from stress and anxiety following an attack on members of their community, largely due to fears of copycat attacks. Yet, empirical evidence for a connection between trigger events and hate-crime related stress has been scarce. Most studies have also limited their samples to members of the target group, rendering them unable to assess how these attacks may “spill over” and affect members of other marginalized groups. In this study, we contribute to this growing body of research on the effects of hate crime on the health and wellbeing of minority group members by evaluating the impact of the Unite the Right rally and vehicle attack in Charlottesville, Virginia on stress and hate crime victimization not just among African-Americans but also among Hispanics and Asian Americans. To do this, we use the nationally representative Stress in America survey and an unexpected event during survey design to identify the causal effect. We find that Black Americans, but not Hispanic or Asian Americans, report greater stress about personal hate crime victimization after the rally.

Correspondingly, we find a significant two-week increase in anti-Black hate crimes following the rally, but no corresponding increase in attacks targeting Hispanics or Asians. It is thus likely that Black respondents reported more stress in response to an actual rise in anti-Black hate crimes following the rally, as shown in the FBI dataset we studied here. This would also explain why rates of stress did not significantly differ among Hispanic and Asian respondents, whose communities were not targeted more frequently after the attack. These results echo those of Wickes et al. (2017), who find that respondents in an Australian sample can “see” hate crime in their communities. Our result goes further, suggesting that people do not only “see” the rate of hate crimes (and specifically those targeting their specific group), but they can also “feel” hate crimes in the form of heightened stress. This finding offers support for the “in terrorem” effect proposed by Perry and Alvi (2012), and thus also for the justification for harsher punishments for hate crime offenders. Episodes of intergroup violence like the events in Charlottesville do seem to have a negative effect beyond the immediate victims of the crime, although this effect may only extend to members of the primary target group.

However, it is also worth noting that this effect may be short-lived. We find that the rally had a significant impact on Black respondents' hate crime-related stress within one week of the event, and a significant impact on anti-Black hate crimes only in the 2 weeks following the event. This also aligns with Google Trends data shown in Figure 2, which suggest that the event remained salient in public discourse for ~1 week. Nevertheless, even acute exposure to stress may have long-term health consequences. Minority group members are rarely exposed to only a single discriminatory event, and the stress from each of these instances can accumulate over the life course into serious health consequences (Pearlin et al., 2005). Thus, the constant threat of hate crime as well as the stress of past exposures may be one factor contributing to chronic racial disparities in overall levels of stress in the U.S. (Sternthal et al., 2011).

Why did other racial groups not experience any significant increase in hate-crime related stress after the events in Charlottesville? We expected that Asian-Americans and Hispanics might also have felt threatened by the rally, given that attacks on one minority group has been linked to creating interminority solidarity (Pérez et al., 2023) and that white supremacists, who primarily targeted African-Americans, would also be seen as prejudiced against other minority groups as well (Chaney and Forbes, 2023). While the Unite the Right rally expressed hatred of a variety of minority racial, ethnic, and religious groups, anti-Black rhetoric was especially prevalent, including prominently displayed symbols of the Ku Klux Klan and the Confederacy. Furthermore, the rally itself was spurred by a debate about the removal of a Confederate statue, thus putting America's history of slavery at the heart of public discourse. The geographic context of the rally in a state where slavery was once legal may also contribute to this impression. In this way, the event may be particularly threatening to Black Americans relative to other ethnic and racial groups. Thus, the results we find here may be specific to the event we studied, and other events may still yield significant spillover effects.

While our analysis meaningfully explores responses to the events in Charlottesville, it nonetheless has its limitations. Most notably, our survey data did not allow us to investigate how the attack affected religious minority groups like Jews and Muslims. Furthermore, the survey data did not allow us to examine the role of contextual factors, such as the racial composition of the region (Kros et al., 2023), which may influence stress levels and subsequent health and wellbeing outcomes among minority group members when hate crimes occur. Compared to other more targeted hate crimes, the Charlottesville rally may have been a particularly likely case for “spillover effects” due to the broad expressions of hate at the rally. These effects may be less common in more targeted events, such as the clearly anti-Asian 2021 spa shooting in Atlanta. Future work should investigate a broader range of trigger events to determine whether these results generalize to other populations and contexts.

If these results are indeed generalizable to other instances of violence, they do not bode well for the U.S. A heated and polarized political climate in which politicians often demonize immigrants and minorities has coincided with an increase in violence targeting these groups (Bilewicz and Soral, 2020; Card et al., 2022). Our results suggest that attacks like those in Atlanta, Buffalo, and El Paso will only yield increased violence against minority communities, and that members of these communities are likely to experience greater stress as a result. These findings suggest that future attacks on members of a specific minority group or highly publicized white supremacist activity should be met with concern and support for members of the targeted community in the weeks following the attack.

## Data availability statement

The data analyzed in this study is subject to the following licenses/restrictions: the Stress in America dataset analyzed for this study can be found in the ICPSR at: https://doi.org/10.3886/ICPSR37288.v1. Additional information on the timing of survey completion must be requested from the American Psychological Association. Requests to access these datasets should be directed to the American Psychological Association, public.affairs@apa.org.

## Ethics statement

Ethical approval was not required for the study involving humans in accordance with the local legislation and institutional requirements. Written informed consent to participate in this study was not required from the participants or the participants' legal guardians/next of kin in accordance with the national legislation and the institutional requirements.

## Author contributions

JH: Writing – review & editing, Writing – original draft, Visualization, Formal analysis. JG: Project administration, Writing – review & editing, Writing – original draft, Supervision, Methodology, Data curation, Conceptualization.

## Footnotes

^1^A later spike in anti-Black hate crimes in late September appears to be unrelated to the rally.

^2^Drakulich et al. (2022) also suggest that hate crimes targeting the White majority have a fundamentally different meaning than crimes targeting minority groups and thus require different theoretical explanations. Therefore, we avoid combining anti-White and anti-minority hate crimes in our analysis.

^3^While scholarship increasingly uses the term “Latine” rather than “Hispanic”, we adopt this terminology in keeping with the language used in the 2017 Stress in America survey.

^4^We thank the Stress in America survey team for providing us with additional information about the date of survey completion, which is not available in the public data set.
