# Supplementary material for: The shadow of fear: hate crime victimization and stress after Charlottesville
Source: Front Psychol. 2024 Jun 27;15:1384470. doi: 10.3389/fpsyg.2024.1384470 (PMC11236755; doi:10.3389/fpsyg.2024.1384470)
Supplement: Supplementary file 1 [file Data_Sheet_1.pdf]

frontiers

Supplementary Material

APPENDIX

Hate Crime Analysis

To test whether the differences seen in Figure 1 are statistically significant, we next run a regression analysis. To address concerns about uneven reporting between departments, we restrict our sample to include only departments that have consistently reported at least one hate crime to the UCR in the 3rd quarter (July-September) of each of the years 2012 to 2017. We restrict our analysis to one quarter in order to account for the seasonality of crime prevalence, which tends to peak in the summer months. Selecting only consistent reporters reduces the sample significantly, leaving us with only 2% of the departments reporting in this period (or 1% of all departments in the dataset) and 33% of the hate crime reports. Our analytical sample thus includes 3,418 hate crimes reported to 64 police departments located in 23 states and the District of Columbia. These departments are primarily located in large metropolitan areas with larger numbers of reported cases. Descriptive statistics for our sample can be found in Table S1. While this sample is not representative of the entire U.S., we argue that these restrictions are necessary due to uneven reporting. Without these restrictions, it is impossible to determine whether a report of zero hate crimes is due to a lack of hate crimes or to a lack of reporting. It is also important to note that our dataset does not include crimes from the Charlottesville area, thus any hate crimes reported at the rally itself will not be counted in our totals. The distribution of hate crimes by month in these consistently reporting police departments is shown in Figure S1; although there are more hate crimes in the year 2017 than in previous years, there does not appear to be a strong upward trend over the term studied here.

Table S1. Statistics by department for consistently reporting agencies (N = 64).

	Number
**Region**	
Midwest	9
Northeast	15
South	19
West	21
**Community size**	
Cities from 0 to 49,999	3
Cities from 50,000 to 99,999	9
Cities from 100,000 to 249,999	12
Cities from 250,000 to 499,999	9
Cities 500,000+	20
Metro area counties 100,000+	11
**Mean annual reported hate crimes**	
Total	8.90
Anti-Black	2.12
Anti-Latine	0.54
Anti-Asian	0.14

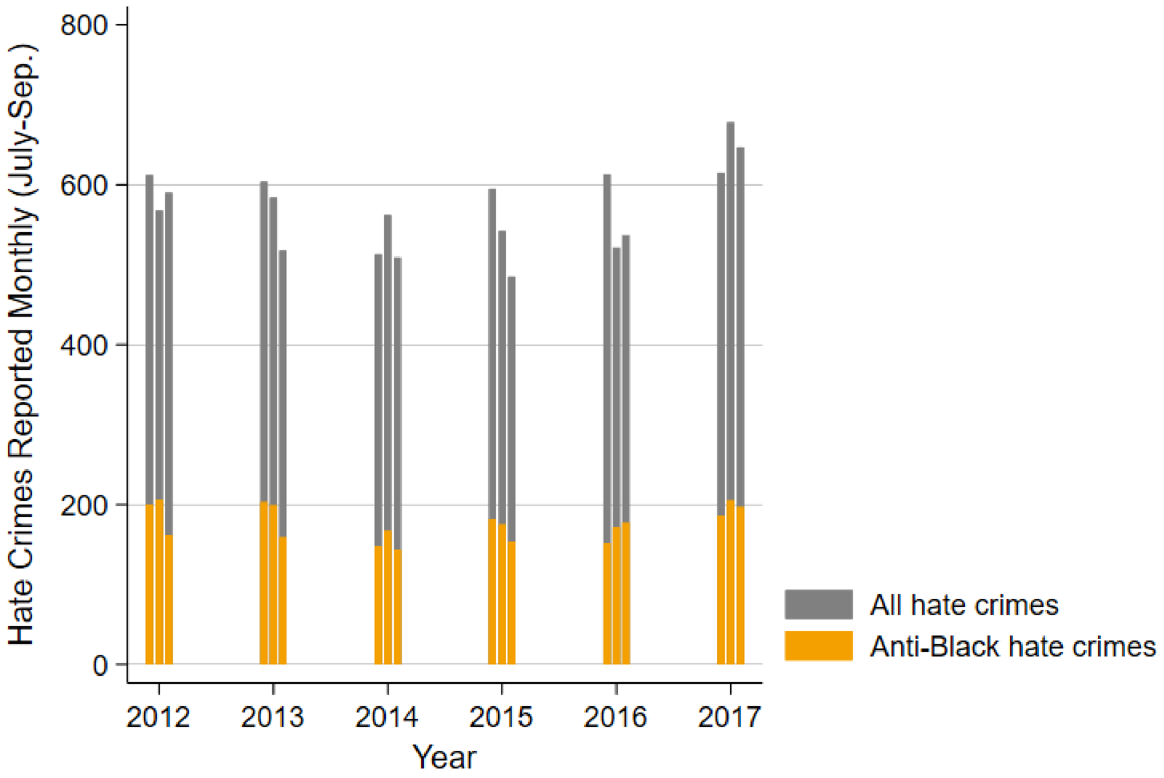

Figure S1. Hate crimes reported by month in consistently reporting police departments, 2012-2017. Source: FBI UCR data.

We employ a difference-in-differences design that compares the difference in crimes in the two weeks before and after the rally on August 12, 2017 with the difference in crimes on the same date in the previous five years. As an outcome variable, we group the number of hate crimes reported in our sample of communities by police department, target group, week, and year, resulting in a number of crimes against each target group per week per jurisdiction. Importantly, the descriptive results shown in Figure 1 do not suggest any violation of the equal trends assumption, given that the number of hate crimes overall and the number of anti-Black hate crimes were fairly level before the rally. We focus on three target groups: Blacks, Latines, and Asians. As our outcome variable is a count of crimes, we use Poisson regression. We also include fixed effects for police department as the number of crimes varies significantly by jurisdiction. For robustness, we also include results from random effects models.

Results from this regression are shown in Table S2. We present estimates for the effect of the date August 12 regardless of year (the date of the rally), the year 2017 (the year of the rally), and the interaction of these two on the reported number of hate crimes targeting Black (Models 1 and 4), Latine (Models 2 and 5), and Asian victims (Models 3 and 6) at the agency level. Both fixed-effects (Models 1-3) and random-effects (Models 4-6) models find a significant positive effect on anti-Black hate crimes following the Unite the Right rally, but no significant effects on anti-Latine or anti-Asian hate crimes. The only other significant value is a negative effect of the date August 12 on anti-Black hate crimes, indicating that such crimes are typically more prevalent in early rather than late August. The lack of significant values for the year 2017 indicates that this year is not significantly different than other years with respect to the number of hate crimes against these three groups.

Table S2. Reported weekly hate crime incidents by agency, 2012-2017 (July 28-August 27).

	FE			RE		
	(1) Anti-Black	(2) Anti-Latine	(3) Anti-Asian	(4) Anti-Black	(5) Anti-Latine	(6) Anti-Asian
After August 12	-0.30*	0.15	0.32	-0.30*	0.15	0.32
	(0.14)	(0.35)	(0.52)	(0.14)	(0.35)	(0.52)
Year 2017	-0.34	0.46	1.14	-0.34	0.46	1.14
	(0.25)	(0.58)	(0.91)	(0.25)	(0.58)	(0.91)
After Charlottesville	0.82*	0.01	-1.93	0.82*	0.01	-1.93
	(0.32)	(0.74)	(1.56)	(0.32)	(0.74)	(1.56)
Constant				-1.74***	-3.52***	-4.38***
				(0.20)	(0.30)	(0.38)
N	1224	672	336	1536	1536	1536

Note: "After Charlottesville" denotes the interaction of "After August 12" and "Year 2017." Unstandardized regression coefficients with standard errors in parentheses.
*p < .05. **p < .01. ***p < .001.

Table S3. "Moving window" analysis: reported anti-Black hate crime incidents, 2012-2017 (July 28-August 27).

	(1) Weeks 1-2	(2) Weeks 2-3	(3) Weeks 3-4	(4) Weeks 4-5	(5) Weeks 5-6
After August 12	-0.30*	-0.16	-0.19	-0.11	-0.11
	(0.14)	(0.12)	(0.14)	(0.15)	(0.14)
Year 2017	-0.34	-0.34	-0.34	-0.34	-0.34
	(0.25)	(0.25)	(0.25)	(0.25)	(0.25)
After Charlottesville	0.82*	0.48	0.55+	0.39	0.39
	(0.32)	(0.31)	(0.33)	(0.34)	(0.30)
N	1224	1200	1224	1224	1248

Note: "After Charlottesville" denotes the interaction of "After August 12" and "Year 2017."
*p < .05. **p < .01. ***p < .001.

Next, we apply a "moving window" analysis (Legewie, 2013) to test the duration of the rally's effect on anti-Black hate crimes. In this analysis, we keep the control period the same as in our earlier models (i.e., the two weeks before the rally) but move the treatment period farther into the future one week at a time. The results are provided in Table S3. Model 1 corresponds to the fixed-effects model presented in Table S2, with "Week 1" meaning the first full week following Charlottesville. As would be expected given the numbers shown in Figure 1, we find a significant effect only in the first two weeks following the rally. Thus, we confirm that the rally led to a two-week spike in anti-Black hate crimes before returning to normal levels.

Balance Tests

Table S4. Balance tests: all minorities.

	Before Mean	After Mean	Diff. in Means	s.e. Diff.	p-value
**Analytic Sample: N = 442 / 680**					
Race: Black	0.36	0.41	0.04	0.03	0.16
Race: Hispanic	0.43	0.38	-0.05	0.03	0.08
Race: Asian	0.20	0.21	0.01	0.02	0.67
Male	0.33	0.32	0.00	0.03	0.94
Age	43.24	46.92	3.68	0.93	0.00
Education (9 pt)	5.00	5.35	0.34	0.11	0.00
Unemployed	0.09	0.10	0.01	0.02	0.60
Party: Democrat	0.52	0.52	0.00	0.03	0.95
Party: Republican	0.13	0.11	-0.02	0.02	0.34
Party: Independent	0.19	0.16	-0.02	0.02	0.37
Party: Other	0.17	0.21	0.04	0.02	0.12
Perc. Own Race in Zipcode	0.36	0.33	-0.03	0.02	0.14
Sexual Minority	0.06	0.07	0.01	0.01	0.40
**Full Sample: N = 738 / 1256**					
Race: Black	0.40	0.38	-0.02	0.02	0.34
Race: Hispanic	0.38	0.38	-0.01	0.02	0.75
Race: Asian	0.22	0.25	0.03	0.02	0.15
Male	0.35	0.33	-0.02	0.02	0.31
Age	42.55	44.81	2.26	0.72	0.00
Education (9 pt)	5.28	5.49	0.20	0.09	0.03
Unemployed	0.09	0.09	0.00	0.01	0.96
Party: Democrat	0.52	0.50	-0.02	0.02	0.45
Party: Republican	0.13	0.11	-0.02	0.01	0.21
Party: Independent	0.17	0.18	0.00	0.02	0.85
Party: Other	0.18	0.21	0.03	0.02	0.08
Perc. Own Race in Zipcode	0.34	0.31	-0.03	0.01	0.03
Sexual Minority	0.06	0.07	0.00	0.01	0.70

Table S5. Balance tests: Black respondents.

	Before Mean	After Mean	Diff. in Means	s.e. Diff.	p-value
**Analytic Sample: N = 161 / 276**					
Male	0.30	0.30	0.00	0.05	0.95
Age	45.70	51.64	5.94	1.46	0.00
Education (9 pt)	4.98	5.45	0.47	0.18	0.01
Unemployed	0.13	0.11	-0.03	0.03	0.42
Party: Democrat	0.69	0.72	0.03	0.05	0.48
Party: Republican	0.04	0.03	-0.01	0.02	0.64
Party: Independent	0.16	0.13	-0.03	0.03	0.41
Party: Other	0.12	0.12	0.01	0.03	0.87
Perc. Own Race in Zipcode	0.40	0.39	-0.01	0.03	0.67
Sexual Minority	0.04	0.06	0.02	0.02	0.27
**Full Sample: N = 295 / 475**					
Male	0.34	0.28	-0.06	0.03	0.08
Age	44.41	48.36	3.94	1.14	0.00
Education (9 pt)	5.11	5.44	0.33	0.14	0.02
Unemployed	0.11	0.09	-0.02	0.02	0.41
Party: Democrat	0.71	0.70	-0.01	0.03	0.86
Party: Republican	0.04	0.03	-0.01	0.01	0.55
Party: Independent	0.13	0.14	0.01	0.03	0.69
Party: Other	0.13	0.13	0.00	0.03	0.88
Perc. Own Race in Zipcode	0.38	0.38	0.00	0.02	0.98
Sexual Minority	0.04	0.05	0.01	0.02	0.62

Table S6. Balance tests: Hispanic respondents.

	Before Mean	After Mean	Diff. in Means	s.e. Diff.	p-value
**Analytic Sample: N = 192 / 260**					
Male	0.30	0.30	0.00	0.04	0.99
Age	40.76	45.48	4.72	1.45	0.00
Education (9 pt)	4.46	4.90	0.44	0.18	0.01
Unemployed	0.08	0.11	0.03	0.03	0.32
Party: Democrat	0.45	0.40	-0.04	0.05	0.35
Party: Republican	0.16	0.17	0.01	0.04	0.83
Party: Independent	0.19	0.19	0.00	0.04	0.90
Party: Other	0.20	0.23	0.03	0.04	0.43
Perc. Own Race in Zipcode	0.40	0.36	-0.04	0.03	0.09
Sexual Minority	0.07	0.08	0.01	0.03	0.65
**Full Sample: N = 282 / 471**					
Male	0.33	0.34	0.01	0.04	0.80
Age	41.02	44.03	3.01	1.15	0.01
Education (9 pt)	4.92	4.98	0.06	0.14	0.66
Unemployed	0.08	0.08	0.00	0.02	0.87
Party: Democrat	0.44	0.40	-0.04	0.04	0.24
Party: Republican	0.18	0.17	-0.02	0.03	0.59
Party: Independent	0.17	0.20	0.03	0.03	0.39
Party: Other	0.21	0.24	0.03	0.03	0.28
Perc. Own Race in Zipcode	0.40	0.35	-0.05	0.02	0.01
Sexual Minority	0.08	0.08	-0.01	0.02	0.80

Table S7. Balance tests: Asian respondents.

	Before Mean	After Mean	Diff. in Means	s.e. Diff.	p-value
**Analytic Sample: N = 89 / 144**					
Male	0.44	0.42	-0.02	0.07	0.75
Age	44.17	40.50	-3.67	1.96	0.06
Education (9 pt)	6.22	5.97	-0.26	0.24	0.27
Unemployed	0.03	0.07	0.04	0.03	0.25
Party: Democrat	0.35	0.33	-0.01	0.06	0.82
Party: Republican	0.22	0.16	-0.06	0.05	0.22
Party: Independent	0.24	0.19	-0.05	0.05	0.38
Party: Other	0.19	0.32	0.13	0.06	0.03
Perc. Own Race in Zipcode	0.17	0.17	0.00	0.02	0.89
Sexual Minority	0.06	0.06	0.00	0.03	0.98
**Full Sample: N = 161 / 310**					
Male	0.41	0.39	-0.02	0.05	0.68
Age	41.80	40.56	-1.24	1.46	0.40
Education (9 pt)	6.24	6.33	0.09	0.18	0.62
Unemployed	0.06	0.08	0.02	0.03	0.33
Party: Democrat	0.31	0.35	0.04	0.05	0.34
Party: Republican	0.20	0.15	-0.06	0.04	0.12
Party: Independent	0.26	0.21	-0.05	0.04	0.18
Party: Other	0.22	0.29	0.07	0.04	0.12
Perc. Own Race in Zipcode	0.17	0.16	-0.01	0.02	0.60
Sexual Minority	0.06	0.07	0.01	0.02	0.63

Placebo Tests

Table S8. Stressed about personally being affected by hate crime: placebo dates. *Note*: *$p < .05$. **$p < .01$. ***$p < .001$.

	(1)	(2)
Placebo 1: Aug. 7 Cutoff Date	-0.07<br>(0.04)	
Placebo 2: August 27 cutoff date		0.03<br>(0.08)
Black	(ref.)	(ref.)
Hispanic	-0.02<br>(0.05)	0.03<br>(0.05)
Asian	-0.05<br>(0.06)	-0.07<br>(0.06)
Placebo 1 × Hispanic	0.06<br>(0.06)	
Placebo 1 × Asian	0.08<br>(0.07)	
Male	0.01<br>(0.03)	-0.04<br>(0.04)
Age	-0.00<br>(0.00)	-0.00*<br>(0.00)
Education (9 pt)	-0.00<br>(0.01)	0.02*<br>(0.01)
Unemployed	0.03<br>(0.06)	0.02<br>(0.07)
Not in labor force	-0.02<br>(0.03)	-0.03<br>(0.04)
Party: Republican	-0.09*<br>(0.04)	-0.13**<br>(0.05)
Party: Independent	-0.03<br>(0.04)	0.01<br>(0.05)
Party: Other	-0.04<br>(0.04)	-0.02<br>(0.05)
Perc. Own Race in Zipcode	-0.07<br>(0.05)	-0.07<br>(0.07)
Sexual minority	0.07<br>(0.06)	0.20*<br>(0.09)
Placebo 2 × Hispanic		-0.07<br>(0.10)
Placebo 2 × Asian		-0.13<br>(0.11)
Constant	0.29***<br>(0.06)	0.20*<br>(0.09)
N	738	434

Robustness Tests

Table S9. Stressed about personally being affected by hate crime: full sample.

	(1)	(2)	(3)
After Charlottesville	0.01	0.05	0.05^{+}
	(0.02)	(0.03)	(0.03)
Black		(ref.)	(ref.)
Hispanic		-0.01	0.01
		(0.03)	(0.03)
Asian		-0.02	-0.01
		(0.03)	(0.04)
After $\times$ Hispanic		-0.04	-0.05
		(0.04)	(0.04)
After $\times$ Asian		-0.06	-0.07
		(0.04)	(0.04)
Male			0.00
			(0.02)
Age			-0.00^{*}
			(0.00)
Education (9 pt)			-0.00
			(0.00)
Unemployed			-0.01
			(0.03)
Not in labor force			-0.03^{+}
			(0.02)
Party: Republican			-0.10^{***}
			(0.02)
Party: Independent			-0.03
			(0.02)
Party: Other			-0.06^{**}
			(0.02)
Perc. Own Race in Zipcode			-0.07^{*}
			(0.03)
Sexual minority			0.11^{**}
			(0.04)
Constant	0.15^{***}	0.15^{***}	0.26^{***}
	(0.01)	(0.02)	(0.04)
N	1994	1994	1994

Note: **p* $<$ .05. ***p* $<$ .01. ****p* $<$ .001.

Table S10. Stressed about personally being affected by hate crime: no Republicans.

	(1)	(2)	(3)
After Charlottesville	0.03<br>(0.02)	0.10**<br>(0.04)	0.11**<br>(0.04)
Black		(ref.)	(ref.)
Hispanic		0.05<br>(0.04)	0.04<br>(0.04)
Asian		0.05<br>(0.05)	0.06<br>(0.05)
After × Hispanic		-0.11*<br>(0.05)	-0.11*<br>(0.05)
After × Asian		-0.12^{+}<br>(0.06)	-0.13*<br>(0.06)
Male			0.00<br>(0.03)
Age			-0.00<br>(0.00)
Education (9 pt)			-0.02*<br>(0.01)
Unemployed			-0.02<br>(0.04)
Not in labor force			-0.04<br>(0.03)
Party: Independent			-0.05<br>(0.03)
Party: Other			-0.07**<br>(0.03)
Perc. Own Race in Zipcode			-0.08^{+}<br>(0.04)
Sexual minority			0.08<br>(0.05)
Constant	0.14***<br>(0.02)	0.11***<br>(0.03)	0.29***<br>(0.06)
N	990	990	990

Note: **p* $<$ .05. ***p* $<$ .01. ****p* $<$ .001.

Table S11. Stressed about personally being affected by hate crime: including White respondents.

	(1)	(2)	(3)
After Charlottesville	0.04**	0.02	0.01
	(0.02)	(0.02)	(0.02)
White		(ref.)	(ref.)
Black		0.07*	-0.01
		(0.03)	(0.03)
Hispanic		0.09**	0.02
		(0.03)	(0.03)
Asian		0.08*	0.00
		(0.04)	(0.04)
After × Black		0.07+	0.08*
		(0.04)	(0.04)
After × Hispanic		-0.03	-0.02
		(0.04)	(0.04)
After × Asian		-0.04	-0.04
		(0.05)	(0.05)
Male			0.02
			(0.02)
Age			-0.00*
			(0.00)
Education (9 pt)			-0.01
			(0.00)
Unemployed			-0.01
			(0.04)
Not in labor force			-0.04*
			(0.02)
Party: Republican			-0.06**
			(0.02)
Party: Independent			-0.05*
			(0.02)
Party: Other			-0.06*
			(0.02)
Perc. Own Race in Zipcode			-0.11***
			(0.03)
Sexual minority			0.07+
			(0.04)
Constant	0.10***	0.05***	0.28***
	(0.01)	(0.01)	(0.05)
N	1661	1661	1661

Note: *$p < .05$. **$p < .01$. ***$p < .001$.

Table S12. Stressed about hate crime as a national issue.

	(1)	(2)	(3)
After Charlottesville	0.12***	0.25***	0.24***
	(0.03)	(0.05)	(0.05)
Black		(ref.)	(ref.)
Hispanic		0.06	0.07
		(0.05)	(0.05)
Asian		0.03	0.09
		(0.06)	(0.07)
After × Hispanic		-0.20**	-0.19**
		(0.07)	(0.07)
After × Asian		-0.22**	-0.22**
		(0.08)	(0.08)
Male			-0.16***
			(0.03)
Age			0.00
			(0.00)
Education (9 pt)			-0.01+
			(0.01)
Unemployed			-0.04
			(0.05)
Not in labor force			-0.00
			(0.03)
Party: Republican			-0.12*
			(0.05)
Party: Independent			-0.05
			(0.04)
Party: Other			-0.05
			(0.04)
Perc. Own Race in Zipcode			0.02
			(0.06)
Sexual minority			0.17**
			(0.06)
Constant	0.36***	0.32***	0.40***
	(0.02)	(0.04)	(0.07)
N	1122	1122	1122

Note: **p* < .05. ***p* < .01. ****p* < .001.

Table S13. Entropy balancing results: stressed about personally being affected by hate crime, by race of respondent.

	(1) Blacks	(2) Hispanics	(3) Asians
After Charlottesville	0.10**	0.00	-0.06
	(0.04)	(0.03)	(0.06)
Constant	0.11***	0.13***	0.18***
	(0.03)	(0.03)	(0.05)
N	437	452	233

Note: **p* $<$ .05. ***p* $<$ .01. ****p* $<$ .001.

Falsification Tests

Table S14. Respondents reporting no stress about money (Model 1) or the economy (Model 2) before and after Charlottesville, by race.

	(1)<br>No stress about money	(2)<br>No stress about the economy
After Charlottesville	-0.05<br>(0.04)	-0.06<br>(0.04)
Black	(ref.)	(ref.)
Hispanic	-0.04<br>(0.04)	-0.09*<br>(0.05)
Asian	-0.09*<br>(0.04)	-0.15**<br>(0.05)
After × Hispanic	0.02<br>(0.05)	0.06<br>(0.06)
After × Asian	0.04<br>(0.05)	0.08<br>(0.06)
Male	0.08***<br>(0.02)	0.05+<br>(0.03)
Age	0.00***<br>(0.00)	0.00*<br>(0.00)
Education (9 pt)	-0.00<br>(0.01)	-0.00<br>(0.01)
Unemployed	-0.04<br>(0.03)	-0.06<br>(0.04)
Not in labor force	0.00<br>(0.02)	0.06*<br>(0.03)
Party: Republican	-0.02<br>(0.03)	-0.02<br>(0.04)
Party: Independent	0.02<br>(0.03)	0.02<br>(0.03)
Party: Other	0.05+<br>(0.03)	0.04<br>(0.03)
Perc. Own Race in Zipcode	-0.03<br>(0.04)	-0.04<br>(0.05)
Sexual minority	-0.08**<br>(0.03)	-0.05<br>(0.05)
Constant	0.06<br>(0.05)	0.20**<br>(0.06)
N	1122	1122

Note: *$p < .05$. **$p < .01$. ***$p < .001$.

Compliance Tests

Table S15. Respondents reporting that the current political climate is a significant stressor (4-point Likert scale), by race.

	(1)
After Charlottesville	0.32** (0.11)
Black	(ref.)
Hispanic	0.35** (0.12)
Asian	0.29* (0.14)
After × Hispanic	-0.21 (0.15)
After × Asian	-0.26 (0.17)
Male	-0.00 (0.07)
Age	0.00 (0.00)
Education (9 pt)	0.02 (0.02)
Unemployed	-0.04 (0.11)
Not in labor force	0.03 (0.07)
Party: Republican	-0.42*** (0.10)
Party: Independent	-0.28** (0.09)
Party: Other	-0.39*** (0.09)
Perc. Own Race in Zipcode	0.04 (0.12)
Sexual minority	0.02 (0.13)
Constant	2.31*** (0.17)
N	1122

Note: *$p < .05$. **$p < .01$. ***$p < .001$.
